# Insight into Inhibitory Mechanism of PDE4D by Dietary Polyphenols Using Molecular Dynamics Simulations and Free Energy Calculations

**DOI:** 10.3390/biom11030479

**Published:** 2021-03-23

**Authors:** Veronika Furlan, Urban Bren

**Affiliations:** 1 Faculty of Chemistry and Chemical Engineering, University of Maribor, Smetanova 17, SI-2000 Maribor, Slovenia; veronika.furlan@um.si; 2 Faculty of Mathematics, Natural Sciences and Information Technologies, University of Primorska, Glagoljaška 8, SI-6000 Koper, Slovenia

**Keywords:** curcumin, 6-gingerol, capsaicin, resveratrol, phosphodiesterase 4D, molecular docking, molecular dynamics simulations, binding free energy calculations, Alzheimer’s disease

## Abstract

Phosphodiesterase 4 (PDE4), mainly present in immune, epithelial, and brain cells, represents a family of key enzymes for the degradation of cyclic adenosine monophosphate (cAMP), which modulates inflammatory response. In recent years, the inhibition of PDE4 has been proven to be an effective therapeutic strategy for the treatment of neurological disorders. PDE4D constitutes a high-interest therapeutic target primarily for the treatment of Alzheimer’s disease, as it is highly involved in neuroinflammation, learning ability, and memory dysfunctions. In the present study, a thorough computational investigation consisting of molecular docking, molecular dynamics (MD) simulations, and binding free energy calculations based on the linear response approximation (LRA) method was performed to study dietary polyphenols as potential PDE4D inhibitors. The obtained results revealed that curcumin, 6-gingerol, capsaicin, and resveratrol represent potential PDE4D inhibitors; however, the predicted binding free energies of 6-gingerol, capsaicin, and resveratrol were less negative than in the case of curcumin, which exhibited the highest inhibitory potency in comparison with a positive control rolipram. Our results also revealed that the electrostatic component through hydrogen bonding represents the main driving force for the binding and inhibitory activity of curcumin, 6-gingerol, and resveratrol, while the van der Waals component through shape complementarity plays the most important role in capsaicin’s inhibitory activity. All investigated compounds form hydrophobic interactions with residues Gln376 and Asn602 as well as hydrogen bonds with nearby residues Asp438, Met439, and Ser440. The binding mode of the studied natural compounds is consequently very similar; however, it significantly differs from the binding of known PDE4 inhibitors. The uncovered molecular inhibitory mechanisms of four investigated natural polyphenols, curcumin, 6-gingerol, capsaicin, and resveratrol, form the basis for the design of novel PDE4D inhibitors for the treatment of Alzheimer’s disease with a potentially wider therapeutic window and fewer adverse side effects.

## 1. Introduction

Phosphodiesterase 4 (PDE4) represents a major family of cyclic adenosine monophosphate (cAMP) hydrolyzing enzymes, which are highly expressed in the brain, cardiovascular tissues, smooth muscles, keratinocytes, and immunocytes (including T cells, monocytes, macrophages, neutrophils, dendritic cells, and eosinophils) [1]. The inhibition of PDE4 results in the elevation of intracellular cAMP, which in turn activates protein kinase A (PKA), cyclic nucleotide-gated ion channels, and exchange factor directly activated by cAMP 1 and 2 (Epac1/2) [2]. This leads to the suppression of pro-inflammatory cytokines and the activation of anti-inflammatory cytokines, maintaining a healthy immune balance. Targeting PDE4 has been verified as an effective therapeutic strategy for chronic inflammatory conditions, including asthma, chronic obstructive pulmonary disease (COPD), psoriasis, atopic dermatitis, inflammatory bowel diseases (IBD), rheumatic arthritis (RA), and lupus [3,4]. Moreover, the involvement of PDE4 in the etiology of central nervous system (CNS) diseases, especially Alzheimer’s disease (AD), has received considerable attention in recent years [5].

### 1.1. PDE4 Structural Biology

PDE4 consists of four subtypes, namely, PDE4A, 4B, 4C, and 4D [3]. PDE4 subtypes can be further divided into long, short, and supershort isoforms, depending on their length. The long isoforms of PDE4 display two upstream conserved regions (UCR1 and UCR2) in the N-terminal region, the short isoform carries only UCR2 and the supershort isoform displays a truncated UCR2. UCR regions are involved in the regulation of PDE4 dimerization and catalytic activities [3]. One of the main differences among isoforms lies in their ability to form homodimers. Long isoforms (such as PDE4D3) are functionally dimeric, while short (such as PDE4D2) and super-short isoforms remain monomeric [6].

PDE4D long isoform is considered the best target for cognition improvement. PDE4D UCR2 carries a key residue, Phe196 that is nonconserved and could be, therefore, exploited to induce isoform specificity (Tyr274 in PDE4B). Exploiting the interactions of PDE4D inhibitors with the capping domain UCR2, which can interact with the inhibitor only in the long isoform, could improve the specificity of the inhibition and assist in the development of partial subisoform selective allosteric modulators with reduced side effects [7]. However, to date, the UCR2 domain is mostly not included in the PBD X-ray structures, or the structures in which the UCR2 is shown to cover the catalytic site remain only partial [7,8].

All PDE4 enzymes possess a highly conserved catalytic domain at the C terminus, which contains 300−350 amino acid residues [9]. The active site of PDE4D enzymes can be divided into three subpockets: (1) a divalent metal pocket that interacts with cAMP (M pocket); (2) a Q pocket that forms hydrogen bonds and hydrophobic interactions with bound PDE4 inhibitors; and (3) a solvated S pocket [10,11]. The M-pocket is characterized by two metal ions, Zn^2+^ and Mg^2+^, and is the most conserved among PDE4D enzymes. Zn^2+^ ion is coordinated by two histidines and two aspartates (His330, His366, Asp367, and Asp484), while the Mg^2+^ ion is coordinated by one bridging aspartate (Asp367). The coordination geometries are octahedral, with free coordination positions filled by water molecules and a hydroxyl group bridging the two metal ions in the cAMP-free enzyme. After cAMP binding to PDE4, the bridging hydroxyl group becomes part of the phosphate group in cAMP, which becomes the interacting partner with both the metal ions, suggesting that the hydroxyl group represents the nucleophile in the hydrolysis of the cyclic phosphodiester bond [12]. The subsequent protonation of O3 atom on cAMP by His326 generates the leaving group, as implicated by the hydrogen bond between His326 and the O3 oxygen found in the PDE4–cAMP complexes. The conserved residues in the active sites of all PDE4 enzymes suggest that the proposed catalytic mechanism could be universal for all PDE4 family members [12].

The S-pocket contains polar amino-acid residues and is filled with water molecules. The Q-pocket represents the most important domain for PDE4D inhibition, which can be further divided into two hydrophobic micro-pockets, Q1 and Q2, separated by a glutamine saddle (Gln535) [13].

The C-terminal region contains control region 3 (CR3) composed of a single relatively short α-helix (12 amino acids), which is also involved in the catalytic activity of the PDE4 enzymes. The important residues of the M, S, and Q pockets together with the CR3 sequence of the PDE4D enzyme (PDB id: 3IAD, chain A) are presented in Table 1. The active site subpockets together with the CR3 region, metal ions, and coordinated water molecules of the PDE4D enzyme are visualized in Figure S1 of the Supplementary Materials.

### 1.2. An Overview of Selective PDE4 Inhibitors and Their Side Effects

Most PDE4 inhibitors produce the inhibition of all four subtypes (PDE4A, PDE4B, PDE4C, and PDE4D) together with the increase in the cAMP concentration above normal physiological levels. Three PDE4 inhibitors, namely, roflumilast, apremilast, and crisaborole, have been already approved worldwide for the treatment of COPD [14], psoriatic arthritis [15], and atopic dermatitis [16], respectively. Moreover, PDE4D inhibitor ibudilast is also approved for the treatment of rare child disease Krabbe by the Food and Drug Administration (FDA) and for bronchial asthma by the Japanese authority [17]. Furthermore, the potential applicability of rolipram in improving learning and memory performance is also experimentally supported [18,19].

However, the above-listed nanomolar PDE4 inhibitors bind the active site competitively with cAMP and, therefore, at high concentrations completely inhibit the enzyme activity (nanomolar affinity). Although the traditional approach to PDE4 inhibitor design has demonstrated therapeutic benefits, these inhibitors increase cAMP concentrations above normal physiological levels, leading to side effects. Adverse side effects, such as nausea, emesis, and gastrointestinal disorders, have consequently largely impeded their clinical applications. The observed side effects are mostly connected with the increased neuronal activity within the area postrema in the CNS [20]. Although both PDE4D and PDE4B are expressed in the area postrema, early studies have suggested that the emesis is caused only by the inhibition of PDE4D [21,22]. However, recent studies imply that the inhibition of PDE4D may not be the key factor causing side effects [7]. A possible reason for this might be the inhibition of nontargeted cellular proteins, which cause a disturbance in normal physiological/cellular functions maintained by the cAMP [23,24].

### 1.3. PDE4D as a Therapeutic Target for the Treatment of Alzheimer’s Disease

Alzheimer’s disease (AD) forms one of the most prevalent age-related neurodegenerative disorders associated with neuro-inflammation in conjunction with the loss of memory and cognitive function. The prevalence rate is about 7% for people aged 65 years or older, with double the risk every 5 years. It currently affects 46 million people worldwide, which is estimated to increase to 131.5 million by 2050 [25]. Neuroinflammation in AD is initiated by deposits of amyloid-β (Aβ) fragments and neurofibrils together with the overproduction of reactive oxygen species (ROS). The inhibition of PDE4 enzymes represents one of the most promising therapeutic strategies for AD treatment. Of the four PDE4 subtypes, PDE4D is especially involved in neuroinflammation, depression, learning ability, and memory dysfunctions, as it is highly expressed in the hippocampus. PDE4D, therefore, represents a therapeutic target of high interest for central nervous system (CNS) diseases, such as AD [19,26,27].

At the molecular level, the PDE4D inhibition leads to the elevation of cAMP levels, resulting in PKA activation and subsequent upregulation of the cAMP response element-binding protein (CREB) in the hippocampus. Phosphorylated CREB promotes neuronal survival through the stimulation of synaptic strengthening and memory formation [19,28]. Moreover, the *in vitro* study of Myeku et al. [29] revealed that increased proteasomal activity through the cAMP/PKA pathway results in reduced tau accumulation, suggesting that PKA induction is also responsible for the enhanced tau clearance leading to improved cognitive functions.

In recent years, the preclinical development of new PDE4 inhibitors for Alzheimer’s disease has gained significant attention in the scientific community. The two main investigated strategies are allosteric modulation [7,30] and the development of inhibitors that show specificity for the PDE4D subtype [31,32,33]. The pioneering work performed by Burgin et al. [7] provided evidence that the difference in the hydrophobic residues Phe196 and Phe201 between PDE4D and PDE4B isoforms, respectively, could assist in the development of allosteric, specific modulators for the PDE4D and PDE4B long isoforms. In this respect, many partial PDE4D inhibitors, namely, BPN14770 [30,34,35], MK-0952 [36], GEBR-32a [32], and chlorbipram [33], have been developed and tested in the clinical trials for the treatment of Alzheimer’s disease. These PDE4D inhibitors relieved AD through the reduction of neuroinflammation and through cognitive enhancement effects with reduced emetic side effects.

Since acetylcholinesterase inhibitors (donepezil, tacrine, rivastigmine, and galantamine) remain the only available symptomatic treatments for AD, more effort and emphasis are required to balance efficacy with minimizing adverse side effects in the development of novel drugs for treating the cause of AD progression.

### 1.4. Natural Polyphenolic Compounds as Potential PDE4D Inhibitors

Natural products represent original resources for modern drug design due to their various beneficial health effects [37]. While the biological effects of natural products may be achieved through interactions with multiple protein targets [38], the regulation of the second messenger cAMP via PDE4 inhibition in the CNS certainly represents a promising strategy for the treatment of neurological diseases. As the key regulator of the cAMP signaling in the CNS, PDE4D most probably serves as a cellular receptor for natural products with observed anti-neuroinflammatory and memory-enhancing effects. Unlike the traditional competitive PDE4D inhibitors that completely inhibit enzymatic activity, natural products are more likely to lower the magnitude of PDE4 inhibition and to maintain cAMP signaling, thereby reducing the potential to cause severe side effects. 

Our efforts have focused on four natural phenolic compounds, curcumin, 6-gingerol, capsaicin, and resveratrol, as potential PDE4D inhibitors. All four polyphenols are known to exhibit a wide range of biological effects, including anti-inflammatory, anticarcinogenic, antibacterial, antiviral, and antifungal activities [38,39,40,41]. Moreover, recent studies have also shown that the listed polyphenols exhibit the potential in the treatment of CNS diseases, especially AD. Curcumin from the plant Curcuma longa (ginger family Zingiberaceae) was reported as a promising compound for the treatment of AD. Curcumin consists of two methoxylated phenolic groups connected by two α,β unsaturated carbonyl groups, which primarily exist in an enol form at physiological pH [42]. Therefore, this form was subjected to our molecular docking and molecular dynamics simulations. On the other hand, its keto form is considered a powerful antioxidant, and the enol form more prone to degradation. A reduction in the amount of Aβ peptide plaque deposition was observed in elderly rats [43] and mice [44,45] with Aβ plaque accumulation after curcumin treatment. Moreover, Abusnina et al. [46] attributed the anti-carcinogenic effects of curcumin to PDE4D inhibition. Furthermore, curcumin demonstrated several beneficial effects in clinical trials, including lowering the plasma Aβ concentrations [47]. 

It was also reported that ginger phenolic compounds, including 6-gingerol, displayed significant inhibitory activity against PDE4D, without nausea and vomiting side effects [48]. Silva et al. [49] *in silico* evaluated the pharmacological properties of capsaicin, extracted from chili peppers, and demonstrated that it probably binds to the peripheral anionic site of the acetylcholinesterase enzyme. Moreover, Xu et al. [50] demonstrated that dietary capsaicin intake prevents AD in Type 2 diabetes. 

**Figure 1:**
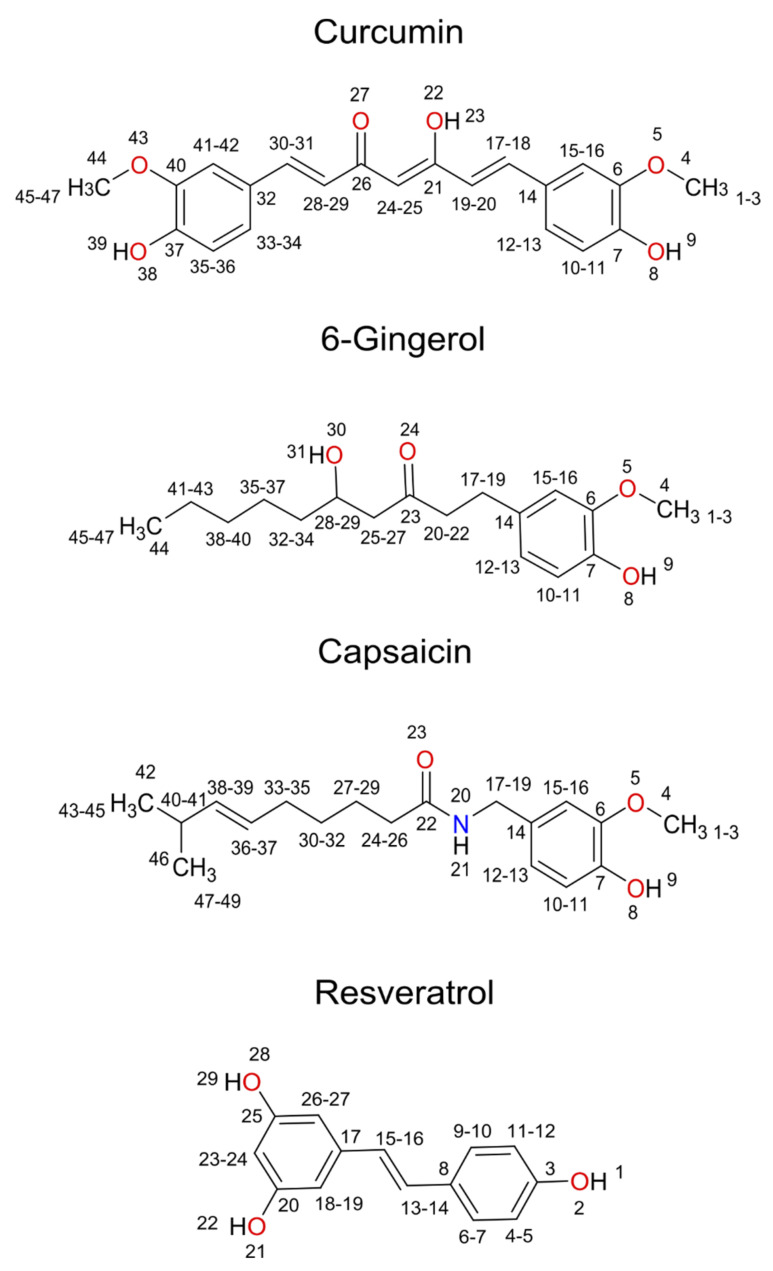
Structural formulas of curcumin, 6-gingerol, capsaicin, and resveratrol with atom identifiers.

In addition, Wang et al. [51] recently found that PDE4D inhibition by resveratrol (40 mg/kg) upregulated signaling pathways associated with neuronal survival (B-cell lymphoma 2 (Bcl-2), CREB, and brain-derived neurotrophic factor (BDNF)) as well as downregulated the neuro-inflammatory interleukins IL-1β and IL-6 in the hippocampus of the amyloid-β1-42-injected mice. Resveratrol was also reported to modulate the PDE4/3-cAMP signaling pathway [52]. Structural formulas of studied polyphenols with atom identifiers are depicted in Figure 1.

The studied natural compounds, therefore, display substantially wider therapeutic windows with reduced side effects (such as emesis) than the earlier, active site-directed PDE4D inhibitors [48]. We believe that an understanding of the structural basis of natural PDE4D inhibitors may enable the development of an alternative strategy for targeting PDE4D in the treatment of AD. Therefore, the objectives of this study are to elucidate the binding modes of four natural products, to determine their mechanism of action, and to predict their inhibitory potency using advanced computer-simulation techniques.

## 2. Computational Methods

*In silico* binding of curcumin in its enol form to PDE4D (PDB ID: 3AID, chain A) was predicted to be favorable by our novel inverse molecular docking protocol [38] performed with the newly developed CANDOCK algorithm [53]. Due to its similarity to the chemical structures of 6-gingerol, capsaicin, and resveratrol, we hypothesized that all four polyphenols may serve as potential PDE4D inhibitors. In order to validate this hypothesis, a computational methodology combining molecular docking, molecular dynamics (MD) simulations, and binding free energy calculations was performed on four systems—PDE4D in complex with curcumin, 6-gingerol, capsaicin, and resveratrol. The studied polyphenolic compounds in complex with PDE4D were obtained with a molecular docking protocol based on the CANDOCK algorithm [53]. Subsequent molecular dynamics simulations and free energy calculations were performed with the Q program package [54] using AMBER ff14 [55] and GAFF [56] force fields as well as the linear response approximation (LRA) method [57].

### 2.1. Molecular Docking

The X-ray crystal structure of PDE4D was obtained from the Protein Data Bank (PDB ID: 3IAD, chain A). The two metal ions (Zn^2+^ and Mg^2+^) in the binding pocket were kept in the crystal structure, as they are conserved among the PDE4 family members [58]. Water molecules coordinating the metal ions were also retained. The 3D structures of investigated natural polyphenols were prepared with Avogadro [59] and subsequently optimized with Gaussian 16 [60] in conjunction with the Hartree-Fock method and 6-31G(d) basis set. To generate the starting models of PDE4D in complex with four natural products, the docking procedure using the CANDOCK algorithm [53] was carried out, and the obtained poses were evaluated by the radial-mean-reduced scoring function at a cutoff radius of 6 Å from each atom of the ligand (RMR6), which was proven to be the best for selecting a crystal-like ligand pose. The CANDOCK algorithm utilizes a hierarchical approach to sample biologically relevant ligand conformations through the reconstruction of ligands from an atomic grid using graph theory and generalized statistical scoring functions. This algorithm also accounts for protein flexibility and ligand interactions with water molecules, metal ions, and cofactors in the binding pocket. The generated poses were evaluated by the RMR6 scoring function. As the crystal structures of curcumin, 6-gingerol, capsaicin, and resveratrol in complex with PDE4D have not yet been experimentally determined, the conformations of studied natural products with the lowest docking score values were subjected to subsequent molecular dynamics (MD) simulations and free-energy calculations to evaluate the stability of protein–ligand interactions.

### 2.2. Preparation of Initial Complexes

After molecular docking, the investigated polyphenolic compounds were extracted from their complexes with PDE4D, and hydrogen atoms were added using Pymol. The obtained ligands were then subjected to full geometry optimization and to subsequent vibrational analysis in the harmonic approximation at the Hartree-Fock (HF) level of theory using 6–31G(d) basis set encoded in the Gaussian 16 program [60]. The restricted electrostatic charge fitting procedure (RESP) was then initiated to reproduce the HF/6–31G(d) calculated electrostatic potential (ESP) surrounding curcumin, 6-gingerol, capsaicin, and resveratrol [61]. Partial charges of chemically equivalent atoms were restricted to the same value. The AMBER ff14 force field was applied for PDE4D, while the missing parameters for the studied natural products, Mg^2+^ and Zn^2+^ ions, were obtained using the general AMBER force field (GAFF) in Antechamber [61]. The missing sidechain atoms of amino acid residues Lys287, His388, and Gln459 were manually added in Pymol, and the missing loop of 17 amino acid residues (Ile576 to Gly593) was structured using Modeller [62]. Topologies and coordinate files necessary for the initiation of molecular dynamics simulations for the bound states were generated with the Qprep5 program.

Systems representing the ligands’ bound states were prepared by constructing a sphere of TIP3P [53] water molecules with a radius of 25 Å positioned at the center of mass of the ligand. A charge of 2+ was assigned to zinc and magnesium ions.

In the area between 22 and 25 Å away from the simulation sphere center, the ionizable residues were treated as neutral entities. All ionizable amino-acid side chains outside of the simulation sphere were modeled as uncharged entities as well [63,64]. The remaining ionizable residues of PDE4D were set to their default protonation states at the physiological pH of 7.4 with the H++ program [65]. His330 and His366, which coordinate the Zn^2+^ ion, were protonated at δ positions [66].

The sum of the total charges in the bound states required the substitution of seven water molecules with seven sodium ions to achieve electroneutrality of the studied complexes. Similarly, the topologies and coordinate files for curcumin, 6-gingerol, capsaicin, and resveratrol using 25Å water spheres in the absence of PDE4D (free states) were prepared for subsequent MD simulations.

### 2.3. Molecular Dynamics Simulations

Each solvated complex was first equilibrated in a series of 12 MD simulations in four parallels using different random seeds with the Qdyn5 module of Q [67,68]. The values of stepsize, temperature, and external bath coupling were gradually increased from 0.01 to 2 fs, 5 to 298,15 K, and 0.1 to 40 fs, respectively. The equilibration phase of 183 ps yielded the starting structure for the production run.

Subsequent molecular dynamics simulations of each studied polyphenol in the bound and free states consist of four 10 ns MD simulations initiated from four independent starting configurations based on different random seeds to representatively cover the available conformational space. Therefore, 32 production trajectories were carried out for 10 ns each in an (N, V, T) ensemble at a temperature of 298,15 K. The integration step was 2 fs, and the SHAKE algorithm was applied to bonds involving hydrogen atoms. The nonbonded interactions were evaluated explicitly for distances shorter than 10 Å. The local reaction field (LRF) method [69] was used to treat the long-range electrostatic interactions for distances beyond the 10 Å cut-off. Energy and coordinate trajectories were stored every 10th and 500th step, respectively, for the subsequent analysis. TIP3P molecules were subjected to the surface constraint all-atom solvent (SCAAS)-type boundary conditions designed to mimic an infinite aqueous solution [70]. Protein atoms protruding beyond the 25 Å simulation sphere were restrained to their initial coordinates, and their nonbonding interactions were turned off [70].

The visualization of coordinate trajectories was performed with Pymol [71] and VMD [72] programs. Analyses of MD trajectories were executed with MD analysis [73] and included calculations of root mean square deviation (RMSD), root mean square fluctuation (RMSF), along with analyses of intermolecular interactions (hydrogen bonds, hydrophobic, and pi–pi interactions). All images were created with Pymol.

### 2.4. Binding Free Energy Calculations with Linear Response Approximation (LRA) Method

Binding free energies of the studied natural polyphenols to PDE4D were calculated with the Qfep5 program from the ligand bound and free states using the linear response approximation (LRA) method.

**Figure 2:**
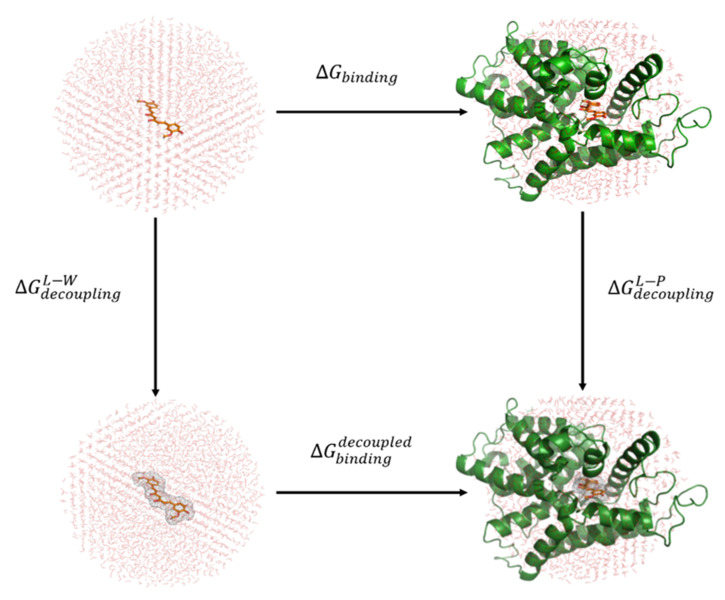
The thermodynamic cycle applied in the linear response approximation (LRA) method. Ligand L is shown in orange color, protein P is portrayed in green color, and red circle W symbolizes water molecules. Gray mesh surrounding the ligand L denotes decoupling–switching off the nonbonded (electrostatic and van der Waals) interactions.

The LRA method of Warshel and co-workers [74] is, similar to the linear interaction energy (LIE) method [75], based on a linear response treating electrostatic interactions and on an empirical term treating the dispersion interactions. The binding of curcumin, 6-gingerol, capsaicin, and resveratrol to PDE4D was, therefore, studied with the help of a thermodynamic cycle depicted in Figure 2.

As can be observed in Figure 2, MD simulations of two physical states—the bound and the free state of an investigated ligand—had to be performed for all four polyphenols. Since free energy represents a state function, the sum of free energy changes depicted in the thermodynamic cycle must be zero:

(1) $$∆G_{binding}+∆G_{decoupling}^{L-P}-∆G_{decoupling}^{L-W}-∆G_{binding}^{decoupled}=0$$

A decoupled ligand has all of its nonbonded interactions switched off; therefore, its binding free energy $∆G_{binding}^{decoupled}$ equals zero. Consequently, Equation (1) can be rearranged as follows:

(2) $$∆G_{binding}=∆G_{decoupling}^{L-W}-∆G_{decoupling}^{L-P}$$

The decoupling terms represent free energy differences accompanying the transfer of a ligand from either aqueous solution (the free state W) or a solvated protein binding site (the bound state P) to a vacuum [76]. The decoupling terms are, therefore, connected with the solvation free energies, which represent free energy differences accompanying the transfer of a ligand from vacuum to either aqueous solution (the free state W) or a solvated protein binding site (the bound state P) and can be further expressed as follows:

(3) $$∆G_{solv}^{L-W}=-∆G_{decoupling}^{L-W}=\alpha \langle V_{vdW}^{L-W}\rangle_{q}+\beta \langle V_{ele}^{L-W}\rangle_{q}$$

(4) $$∆G_{solv}^{L-P}=-∆G_{decoupling}^{L-P}=\alpha \langle V_{vdW}^{L-P}\rangle_{q}+\beta \langle V_{ele}^{L-P}\rangle_{q}$$

Preorganized electrostatics term $\langle V_{ele}^{L-P}\rangle_{0},$ additionally included in the LRA methodology, reflects the electrostatic interaction of a ligand L with its preorganized environment, the geometry and polarization of which are established in the presence of zero partial charges on all ligand atoms [77].

The binding free energy of a ligand L to the protein binding site P can then be expressed as a linear combination of interaction energies presented in Equation (5):

(5) $$∆G_{binding}^{L-P}=\alpha \left(\langle V_{vdW}^{L-P}\rangle_{q}-\langle V_{vdW}^{L-W}\rangle_{q}\right)+\beta \left(\langle V_{ele}^{L-P}\rangle_{q}-\langle V_{ele}^{L-W}\rangle_{q}\right)+\beta \langle V_{ele}^{L-P}\rangle_{0}$$

where $\langle V_{vdW}^{L-P}\rangle_{q}$, $\langle V_{vdW}^{L-W}\rangle_{q}$, $\langle V_{ele}^{L-P}\rangle_{q}$, and $\langle V_{ele}^{L-W}\rangle_{q}$ represent van der Waals and electrostatic interaction energies between the ligand L and its surroundings—either aqueous solution W or solvated protein binding site P—averaged over an ensemble of configurations generated by MD simulations. The subscript q indicates that this ensemble is obtained with the regular partial atomic charges of the ligand. As in the LIE method, the initial value of β was set to 0.5, while the value of α was determined empirically and equals 0.18 [78]. However, Aqvist and Hansson [79] further determined the first set of refined values for the scaling factor β as a function of the chemical nature of the ligand based on the Free Energy Perturbation (FEP) calculations. It was observed that for neutral compounds bearing two or more hydroxyl/polar groups, the β parameter should equal 0.33. According to the authors, this value represents a deviation from the linear response theory, which is directly related to the participation of the ligand in the hydrogen bond network of the aqueous solution.

$\langle V_{es}^{L-P}\rangle_{0}$ represents the average electrostatic interaction energy between the ligand L and its solvated surrounding protein binding site P calculated over an ensemble of configurations generated by MD simulations in which all ligand partial atomic charges are set to zero [77]. In proteins, the binding site dipoles associated with polar groups and ionized residues may already be partially oriented toward the bound ligand with all of its partial atomic charges set to zero. The instantaneous charging of the bound ligand may, therefore, yield favorable electrostatic interactions between the protein and its ligand ($\langle V_{ele}^{L-P}\rangle_{0}<0$). Consequently, it can be claimed that proteins are electrostatically preorganized to accommodate their ligands. The origin of preorganized electrostatics can thus be traced back to the process of protein folding, whereby the protein attains its 3D structure, and its dipoles, as well as ionized residues, become oriented. On the other hand, if the water and the solute geometries remain unchanged during the analogous instantaneous charging process of the solute, the overall interaction energy is zero ($\langle V_{ele}^{L-W}\rangle_{0}=0$). Therefore, it can be said that polar solvents are not electrostatically preorganized to accommodate solute molecules. 

## 3. Results and Discussion

### 3.1. Molecular Docking

Molecular docking was used to explore the interactions of PDE4D with curcumin, 6-gingerol, capsaicin, and resveratrol. The four natural products were docked into the binding site pocket of PDE4D using the CANDOCK algorithm and the generalized statistical scoring function RMR6 [53] to generate initial complexes for subsequent MD simulations as described in the Methods section. After the initial visual inspection, the binding modes were selected based on the lowest docking score values, which are for curcumin, 6-gingerol, capsaicin, and resveratrol in complex with PDE4D presented in Table 2. A redocking procedure of the native PDE4D inhibitor 15X was also performed (PDB ID 3IAD, chain A). The comparison of the native (yellow) and redocked (orange) 15X poses at the active site of PDE4D enzyme is presented in Figure S2 of the Supplementary Materials. The calculated RMSD value between the best-scoring redocked pose and the native pose of the PDE4D inhibitor 15X is lower than 2 Å (0.94 Å), which additionally confirms the validity of the applied molecular docking protocol [80].

The results of the docking study revealed that all investigated polyphenols exhibit acceptable binding affinity to PDE4D (docking score values <−30 arb. units). These results are consistent with the existing experimental data [46,48,49,50,51,52] and support the hypothesis that the studied polyphenolic compounds exhibit an inhibitory effect on PDE4D. Moreover, the docking results provided the first evaluation, which polyphenolic compounds exhibit a stronger binding affinity (lower docking score value) to PDE4D. As can be inferred from Table 1, curcumin and 6-gingerol showed a higher affinity to PDE4D than capsaicin and resveratrol.

### 3.2. RMSD Analysis

**Figure 3:**
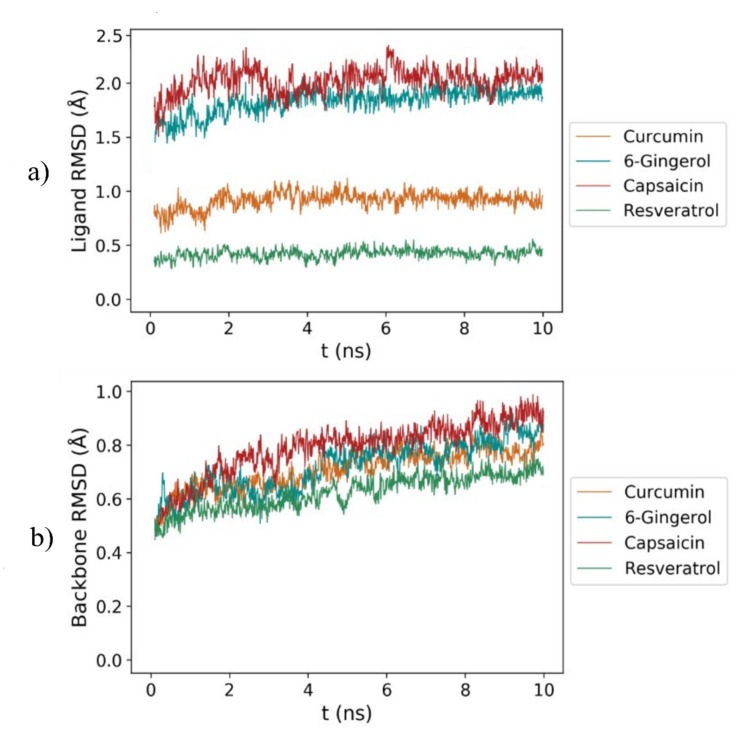
Root mean square deviation (RMSD) curves of (**a**) ligand atomic positions and (**b**) backbone atomic positions throughout 10 *ns* molecular dynamics simulations of four investigated systems: curcumin in complex with PDE4D (orange), 6-gingerol in complex with PDE4D (blue), capsaicin in complex with PDE4D (red), and resveratrol in complex with PDE4D (green).

To investigate the dynamic stability of PDE4D/curcumin, PDE4D/6-gingerol, PDE4D/capsaicin, and PDE4D/resveratrol complexes obtained via molecular docking, four independent 10 ns molecular dynamics simulations were performed for each complex with the Q5 program package. The conformational stability of the protein–ligand complexes was assessed by the root mean square deviation (RMSD) of ligand and protein backbone atoms during the entire production run. The RMSD values of each ligand were calculated with respect to its initial docked configuration after a translational and rotational fit of the protein backbone atoms at the active site of PDE4D. The RMSD values of PDE4D backbone atoms with respect to their initial crystal structure configuration were also calculated after a translational and rotational fit of PDE4D backbone atoms. The RMSD curves of the studied ligands and PDE4D backbone atoms are presented in Figure 3a,b, respectively. The average RMSD curves of PDE4D active site atomic positions in the radius of 6 Å around the four investigated ligands throughout four 10 ns molecular dynamics simulation production runs are also provided in Figure S3 of the Supplementary Materials.

The average ligand and backbone RMSD values of all production runs are further collected in Table 3.

The RMSD values of the studied ligands bound to PDE4D were monitored during MD simulation production runs, which yielded stable RMSD curves, indicating that the simulated systems were well equilibrated and that the equilibration protocol was appropriate for the PDE4D system. RMSD curves for curcumin, 6-gingerol, capsaicin, and resveratrol stabilized at average values of 0.92 ± 0.08, 1.74 ± 0.16, 1.99 ± 0.14, 0.40 ± 0.04 Å, respectively (Table 1). The obtained results suggest that both the number of phenol groups and the length of the alkyl chain affect the stability of atomic positions in the investigated inhibitors. Resveratrol exhibited the lowest average RMSD values due to its small, rigid structure with three phenol groups. Curcumin displayed slightly higher RMSD values, probably due to its larger size and less rigid structure. On the other hand, 6-gingerol and capsaicin exhibited the highest average RMSD values, due to their long alkyl chains with a high number of rotatable bonds and only a single phenol group. However, all average RMSD values were below 2 Å, indicating stable ligand poses for all MD simulations [81].

Moreover, from Figure 3 and Table 3, it can be observed that RMSD curves for the backbone atoms with all four investigated natural products converged below 1 Å, which indicates that all MD trajectories achieved equilibrium and once again proves that the current MD equilibration protocol was suitable for the PDE4D system. The average RMSD backbone values for curcumin, 6-gingerol, capsaicin, and resveratrol in complex with PDE4D were 0.71 ± 0.08, 0.72 ± 0.11, 0.78 + 8 ± 0.08, and 0.63 ± 0.07 Å, respectively.

All in all, small average deviations of PDE4D backbone atom positions suggest the presence of the polyphenolic ligands as the key element for maintaining PDE4D active site conformation close to the initial crystal structure.

### 3.3. RMSF Analysis

**Figure 4:**
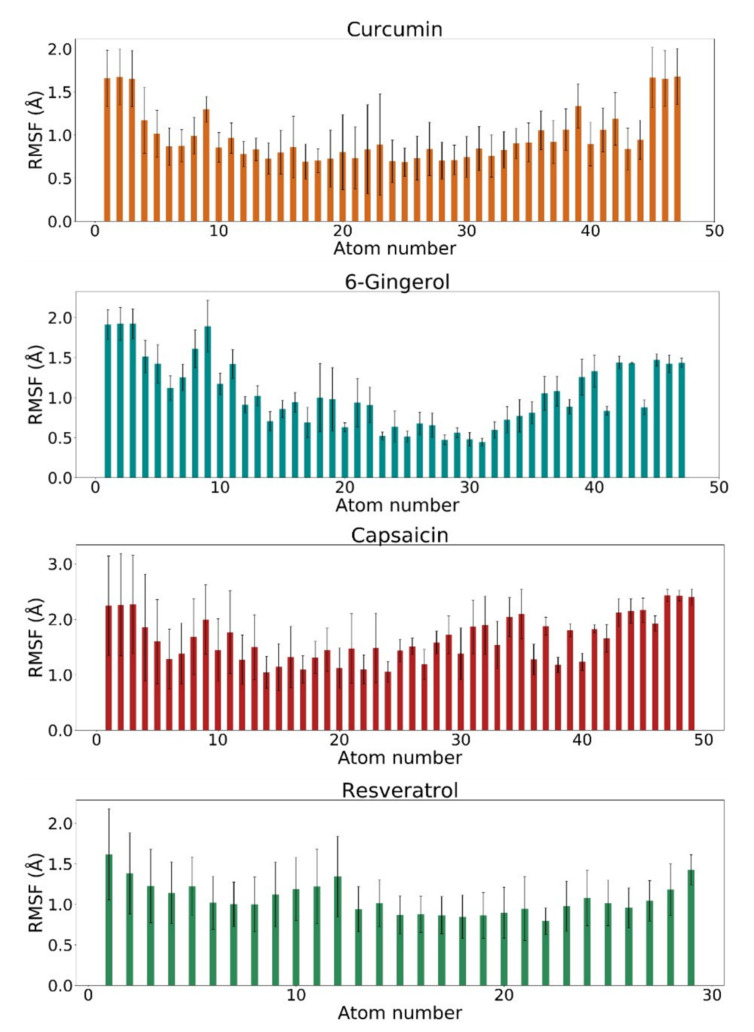
Root mean square fluctuation (RMSF) values of ligand atomic positions throughout molecular dynamics simulation production runs of four investigated systems: curcumin in complex with PDE4D (orange), 6-gingerol in complex with PDE4D (blue), capsaicin in complex with PDE4D (red), and resveratrol in complex with PDE4D (green). The atom numbers correspond to ligand numbering presented in Figure 1. Error bars indicate standard deviations between four independent MD simulations.

Moreover, root mean square fluctuations (RMSF) of the studied ligands were calculated over the course of the MD simulation production runs. The RMSF values of atomic positions of four investigated polyphenols are presented in Figure 4.

The average RMSF graphs of PDE4D active site atomic positions in the radius of 6 Å around the four investigated ligands throughout four 10 ns molecular dynamics simulation production runs are also provided in Figure S4 of the Supplementary Materials. Furthermore, the average RMSF values of ligand atomic positions throughout four independent 10 ns molecular dynamics simulation production runs of the four investigated systems are reported in Table 4.

The conformational stability of curcumin, 6-gingerol, capsaicin, and resveratrol is highlighted by average RMSF values of 0.98 ± 0.09, 1.11 ± 0.14, 1.79 ± 0.12, and 0.97 ± 0.07 Å, respectively, which are in agreement with the previously discussed RMSD values of the ligands (Table 3).

In addition, from Figure 4, it can be observed that the RMSF profiles of the four investigated natural compounds in complex with PDE4D display similar characteristics. Terminal regions of curcumin, 6-gingerol, capsaicin, and resveratrol are more flexible than their middle parts, mainly due to hydrogen atoms at methoxy and phenolic groups or hydrogen atoms at the end of alkyl chains (in the case of capsaicin and 6-gingerol), which are not participating in the binding interactions with PDE4D. All in all, average RMSF values remained below 2 Å, suggesting that the conformations of all ligands remain close to their initial structures.

### 3.4. Binding Patterns of Curcumin, 6-Gingerol, Capsaicin, and Resveratrol to PDE4D

**Figure 5:**
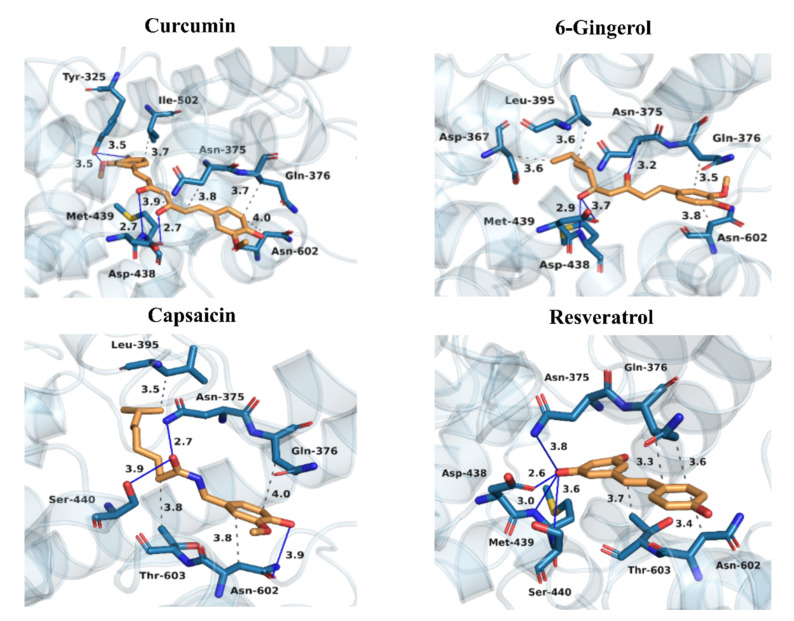
Binding modes of curcumin, 6-gingerol, capsaicin, and resveratrol at the active site of PDE4D. Carbon atoms of the investigated polyphenols are presented in orange and carbon atoms of PDE4D amino-acid residues in light blue color. Oxygen atoms are red, nitrogen atoms dark blue, and sulfur atoms yellow. Hydrophobic interactions are depicted with gray and hydrogen bonds with dark blue lines. All distances are given in Å and represent averages over all MD production run snapshots for each complex. Hydrogen atoms are omitted for clarity.

To reveal the inhibitory mechanisms of four investigated natural products, detailed interactions between them and PDE4D were evaluated using the protein ligand interaction profiler (PLIP) [82]. The results of the PLIP analysis of the four investigated polyphenols bound to PDE4D are depicted in Figure 5, which enables the identification of the key binding amino-acid residues of PDE4D. The occurrences of the most important hydrogen bonds between PDE4D and the studied polyphenols were also monitored according to the geometric criterion used in the PLIP algorithm.

From the binding modes in Figure 5, it can be deduced that curcumin, 6-gingerol, capsaicin, and resveratrol formed hydrophobic interactions with residues Gln376 (3.73, 3.46, 3.96, and 3.56 Å, respectively) and Asn602 (3.95, 3.77, 3.85, and 3.40 Å, respectively). Curcumin was further stabilized through hydrophobic interactions with nearby residues Asn375, Met439, and Ile502 at distances of 3.81, 3.94, and 3.70 Å, respectively. Both capsaicin and 6-gingerol formed additional hydrophobic interactions with Leu395 (3.54 Å and 3.59 Å, respectively), while hydrophobic interactions with Thr603 were observed in the case of capsaicin and resveratrol (3.79 and 3.74 Å, respectively). In addition, 6-gingerol formed a hydrophobic interaction with nearby residue Asp367 (3.64 Å).

Moreover, curcumin, 6-gingerol, and resveratrol formed hydrogen bonds with nearby residues Asp438 (2.73 (96% occurrence), 3.71 (85% occurrence), and 2.62 (86% occurrence) Å, respectively), and Met439 (2,73 (95 % occurrence), 2.94 (88 % occurrence), and 3.61(84% occurrence) Å, respectively), while capsaicin formed a hydrogen bond with the nearby Ser440 (3.91 (78% occurrence) Å). Furthermore, curcumin formed additional hydrogen bonds with Tyr325 (3.53 and 3.54 Å in 87 and 89% of time, respectively), while a hydrogen bond with Asn375 was observed in the case of 6-gingerol, capsaicin, and resveratrol (3.23 (83% occurrence), 2.74 (87% occurrence), and 3.82 (81% occurrence) Å, respectively). In addition, capsaicin formed a hydrogen bond with Asn602 at a distance of 3.90 Å in 86% of time.

The hydrophobic and hydrogen bonding interactions, therefore, play a vital role in stabilizing all four investigated inhibitors in the active pocket. It can be observed that similar amino-acid residues play an important part in the binding of the studied natural products to PDE4D, which confirms that all four ligands occupy the same active site.

Most of the known inhibitors occupy the active site of PDE4 enzymes, primarily the Q pocket, forming interactions with hydrophobic residues, such as Tyr325, Leu395, Leu485, Tyr495, Trp498, Thr499, Ile502, Val531, and Phe506, as well as hydrogen bonds with the Gln535 residue [41]. In our case, the studied natural compounds exhibited a different binding pattern in the M pocket, with very limited interaction with Q1/Q2 pockets and Gln535. The graphical comparison of the binding modes of the studied polyphenols and the native inhibitor 15X as well as the established PDE4D inhibitor rolipram is presented in Figure S5. The interactions with amino-acid residues at the M pocket are, therefore, considered as the most important for the binding of the four studied inhibitors. However, the additional interactions of curcumin with the Q pocket residues Tyr325 and Ile502 might contribute to its high affinity. The shifts of the overall skeleton of this class of natural inhibitors closer to the M pocket may be the consequence of the absent hydrogen bond with Gln535. In addition, our results clearly show that all four inhibitors formed hydrophobic interactions with nonconserved residues in the CR3 region, especially with Asn602. However, it must be noted that the number of phenolic groups and the overall molecular size also influence the activity of the studied inhibitors.

In summary, the binding patterns of the four studied polyphenolic compounds are very similar. All of them formed hydrophobic interactions with residues Gln376 and Asn602 as well as hydrogen bonds with nearby residues Asp438, Met439, and Ser440. Our results, therefore, suggest the key residues for the intermolecular interactions between PDE4D and the studied natural products that should be considered carefully in the structure-based design of novel PDE4D inhibitors. Most of the aforementioned hydrophobic and hydrogen bonding interactions were not observed in the crystal structures of known PDE4 inhibitors. Presented binding modes may, therefore, pave a new way for the discovery of novel PDE4D inhibitors.

### 3.5. Results of Binding Free Energy Calculations with the LRA Method

Our study was further extended with the binding free energy calculations of the investigated polyphenols to the binding pocket of PDE4D. The van der Waals (vdW) and electrostatic (ele) nonbonded interactions were calculated for all four investigated polyphenols with the LRA methodology in order to compare their binding capability. Binding free energies of curcumin, 6-gingerol, capsaicin, and resveratrol to PDE4D calculated by using preoptimized values of empirical coefficients 0.18 (α) and 0.33 (β) [79] of the LRA Equation (5) are assembled in Table 5. For the evaluation of the obtained binding free energies, the experimental binding free energy of a positive control, rolipram, is also provided [83].

The predicted binding free energies for curcumin/PDE4D, 6-gingerol/PDE4D, capsaicin/PDE4D, and resveratrol/PDE4D complexes were calculated using the Qfep5 program. From Table 5, it can be observed that the PDE4D/curcumin complex (−11.03 ± 0.25 kcal/mol) showed a more negative binding free energy than PDE4D/6-gingerol (−6.99 ± 0.39 kcal/mol), PDE4D/capsaicin (−5.24 ± 0.35 kcal/mol), and PDE4D/resveratrol (−4.59 ± 0.41 kcal/mol) complexes, exactly reproducing the order of docking score values from Table 2. Moreover, the calculated binding free energy of curcumin is also slightly lower (by −1.17 kcal/mol) than the experimental binding free energy of the positive control rolipram (−9.86 kcal/mol), which represents the established PDE4D inhibitor [83].

The binding free energies revealed that hydrogen bonding through electrostatic interactions plays the main role in curcumin (−60.66 ± 1.15 kcal/mol), 6-gingerol (−49.96 ± 0.5 kcal/mol), and resveratrol (−52.05 ± 0.65 kcal/mol) binding to PDE4D. For curcumin, the electrostatic term contributing to the binding free energy in Equation (5) is consistently much more negative (up to −22.73 kcal/mol) than for other polyphenols, resulting in the lowest binding free energy among all studied polyphenols (−11.03 ± 0.25 kcal/mol). On the other hand, the least negative van der Waals contribution in the case of resveratrol (−30.55 ± 0.39 kcal/mol) resulted in the highest binding free energy (−4.59 ± 0.41 kcal/mol). On the contrary, nonpolar van der Waals interactions through shape complementarity represent the main driving force for binding and the inhibitory activity of capsaicin (−51.49 ± 1.15 kcal/mol), while electrostatics plays only a minor role (−37.93 ± 0.7 kcal/mol). However, electrostatics remains very important for the proper orientation of all investigated polyphenols in the active site of PDE4D, as indicated by the presence of multiple hydrogen bonds depicted in Figure 5.

In addition, both electrostatic and van der Waals contributions to binding between the investigated polyphenols and PDE4D are favorable, as indicated by lower average protein–ligand interaction energies in complex when compared to free polyphenols in water. The binding site dipoles associated with polar groups and ionized residues are already partially oriented towards all bound ligands with all their partial atomic charges set to zero, which is indicated by favorable preorganized electrostatic interactions between PDE4D and investigated polyphenols ($\langle V_{ele}^{L-P}\rangle_{0}<0$). As a result, both van der Waals/hydrophobic and electrostatic/hydrogen bonding interactions play an important role in the mechanism of inhibition.

## 4. Conclusions

Natural products play a vital role in the development of new drugs because they possess several advantages over conventional synthetic compounds, namely, fewer side effects, lower long-term toxicity, and versatile biological effects. Although natural products may target multiple proteins, PDE4D certainly represents an important target in the treatment of neurological disorders, such as Alzheimer’s disease. However, the natural PDE4D inhibitors are rare, and their crystal structures with PDE4D are unavailable. Therefore, it is sensible to study the interaction mechanisms between the natural products and PDE4D by computational means. In the present study, molecular docking, MD simulations, and binding free energy calculations were performed to investigate the inhibitory mechanisms of four natural products, namely, curcumin, 6-gingerol, capsaicin, and resveratrol against PDE4D. 

The analysis of MD trajectories correlated well with the free energy calculations and facilitated the recognition of important structural features for inhibitory activity. The predicted binding free energies are −11.03 ± 0.25, −6.99 ± 0.39, −5.24 ± 0.35, and −4.59 ± 0.41 kcal/mol for the PDE4D/curcumin, PDE4D/6-gingerol, PDE4D/capsaicin, and PDE4D/resveratrol complexes, respectively, which is fully consistent with the inhibitory potency order predicted by molecular docking. Moreover, the predicted binding free energy of curcumin is also lower than the experimental binding free energy of a positive control rolipram (−9.86 kcal/mol), which confirms its high PDE4D inhibitory potency.

It was also revealed that the electrostatic component through hydrogen bonding represents the main driving force for the binding and inhibitory activity of curcumin, 6-gingerol, and resveratrol, while the van der Waals component through shape complementarity plays the most important role in capsaicin’s inhibitory activity. The binding mechanism of all investigated natural compounds is very similar; however, it significantly differs from the mechanism of known PDE4 inhibitors. All studied compounds form hydrophobic interactions with residues Gln376 and Asn602 as well as hydrogen bonds with nearby residues Asp438, Met439, and Ser440. The interactions with amino-acid residues from the M pocket and CR3 region of PDE4D are, therefore, considered as the most important for the binding of the investigated polyphenols.

The established computational approach consisting of molecular docking, MD simulations, and free energy calculations was proven to be a useful exploratory tool for biological systems important in drug discovery, especially for cases when it is difficult to obtain the crystal structure of protein–ligand complexes. This study also confirms the general applicability of empirical binding free energy methods, such as LRA, to study challenging biomolecular systems, such as PDE4D. We firmly believe that this research will assist in the design and development of novel PDE4D small molecule inhibitors with fewer side effects and a wider therapeutic window for the treatment of Alzheimer’s disease.

## Figures and Tables

**Table 1:** The important residues of M, S, and Q pockets of the catalytic domain, together with the CR3 sequence of PDE4D.

Pocket Name	Features	Important Residues
M-pocket	Contains metal ions Zn^2+^ and Mg^2+^	His330, His366, Asp367, His370, Asn375, Gln376, Leu395, Glu396, Asp438, Met439, Ser440, Asp484
S-pocket	Contains polar residues and water molecules	Gly372, Ser374, Glu505, Arg508, Ser521, Cys524
Q-pocket	Divided into two hydrophobic micro-pockets Q1 and Q2, separated by a glutamine saddle (Gln535)	Q1-pocket: Tyr325, Asn487, Thr499, Tyr495 Q2-pocket: Ile502, Met503, Phe506, Met523, Phe538
CR3	α-helix consisting of 12 amino-acid residues	Gln595, Val596, Ser597, Glu598, Phe599, Ile600, Ser601, Asn602, Thr603, Phe604, Leu605, Asp606

**Table 2:** Docking score values of the best-scored PDE4D–polyphenol complexes obtained with CANDOCK algorithm and RMR6 scoring function.

Ligands	Docking Score Values (Arbitrary Units)
Curcumin	−62.24
6-Gingerol	−50.16
Capsaicin	−41.58
Resveratrol	−30.18

**Table 3:** Average RMSD values of ligand and protein backbone atomic positions throughout four independent 10 ns molecular dynamics simulation production runs of the four investigated systems: curcumin in complex with PDE4D, 6-gingerol in complex with PDE4D, capsaicin in complex with PDE4D, and resveratrol in complex with PDE4D.

MD Simulation Run	Production Run 1	Production Run 2	Production Run 3	Production Run 4	Average of All Runs
Curcumin					
Average ligand RMSD (Å)	0.93	0.87	0.88	0.99	0.92 ± 0.08
Average backbone RMSD (Å)	0.69	0.75	0.68	0.72	0.71 ± 0.08
6-Gingerol					
Average ligand RMSD (Å)	1.53	1.82	1.72	1.90	1.74±0.16
Average backbone RMSD (Å)	0.76	0.68	0.72	0.71	0.72 ± 0.11
Capsaicin					
Average ligand RMSD (Å)	1.95	1.83	2.10	2.08	1.99 ± 0.14
Average backbone RMSD (Å)	0.71	0.82	0.81	0.81	0.78 ± 0.11
Resveratrol					
Average ligand RMSD (Å)	0.47	0.35	0.36	0.43	0.40 ± 0.04
Average backbone RMSD (Å)	0.58	0.63	0.67	0.64	0.63 ± 0.07

The average values of all production runs are presented as mean ± standard deviation.

**Table 4:** Average RMSF values of ligand atomic positions throughout four independent 10 ns molecular dynamics simulation production runs of four investigated systems: curcumin in complex with PDE4D, 6-gingerol in complex with PDE4D, capsaicin in complex with PDE4D, and resveratrol in complex with PDE4D.

MD Simulation Run	Production Run 1	Production Run 2	Production Run 3	Production Run 4	Average of All Runs
Curcumin					
Average ligand RMSF (Å)	1.08	1.07	0.92	0.85	0.98±0.09
6-Gingerol					
Average ligand RMSF (Å)	1.30	0.97	1.07	1.10	1.11±0.14
Capsaicin					
Average ligand RMSF (Å)	1.71	1.91	1.67	1.86	1.79±0.12
Resveratrol					
Average ligand RMSF (Å)	1.00	0.88	0.95	1.05	0.97±0.07

The average values of all production runs are presented as mean ± standard deviation.

**Table 5:** The average electrostatic (ele) and van der Waals (vdW) nonbonded interactions of curcumin, 6-gingerol, capsaicin, and resveratrol in water (the free state W) as well as in complex with PDE4D (the bound state P) along with the corresponding binding free energies.

Energies	$\left\langle V_{vdW}^{L-P}\right\rangle$ (kcal/mol)	$\left\langle V_{vdW}^{L-W}\right\rangle$ (kcal/mol)	$\left\langle V_{ele}^{L-P}\right\rangle$ (kcal/mol)	$\left\langle V_{ele}^{L-W}\right\rangle$ (kcal/mol)	$\langle V_{ele}^{L-P}\rangle_{0}$ (kcal/mol)	$∆G_{binding}^{L-P}$ ** (kcal/mol)
Curcumin						
Production run 1	−53.72	−33.43	−60.10	−40.34	−0.66	−10.83
Production run 2	−52.38	−33.41	−62.07	−40.55	−0.73	−11.24
Production run 3	−52.33	−33.52	−61.04	−39.43	−0.69	−11.22
Production run 4	−52.94	−33.41	−60.43	−40.23	−0.69	−10.85
Average *	−52.84 ± 0.64	−33.44 ± 0.05	−60.66 ± 1.15	−40.14 ± 0.49	−0.69 ± 0.03	−11.03 ± 0.25
6-Gingerol						
Production run 1	−49.25	−24.38	−49.89	−43.84	−0.49	−6.97
Production run 2	−50.71	−24.49	−49.37	−43.16	−0.51	−7.27
Production run 3	−49.11	−25.40	−50.58	−44.17	−0.47	−6.85
Production run 4	−48.15	−24.36	−49.98	−43.53	−0.44	−6.85
Average *	−49.56 ± 1.52	−24.66 ± 0.50	−49.96 ± 0.50	−43.67 ± 0.43	−0.48 ± 0.03	−6.99 ± 0.39
Capsaicin						
Production run 1	−50.33	−28.20	−38.69	−35.67	−0.38	−5.36
Production run 2	−52.33	−27.72	−37.29	−35.81	−0.35	−5.27
Production run 3	−52.62	−27.74	−37.39	−35.76	−0.36	−5.37
Production run 4	−50.67	−28.30	−38.37	−36.70	−0.38	−4.96
Average *	−51.49 ± 1.15	−27.99 ± 0.31	−37.93 ± 0.70	−35.99 ± 0.48	−0.37 ± 0.02	−5.24 ± 0.35
Resveratrol						
Production run 1	−31.64	−16.53	−51.15	−47.18	−0.53	−4.57
Production run 2	−30.18	−16.44	−51.90	−47.04	−0.50	−4.58
Production run 3	−29.80	−16.46	−52.68	−47.61	−0.48	−4.56
Production run 4	−30.58	−16.49	−52.48	−47.59	−0.49	−4.65
Average *	−30.55 ± 0.39	−16.48 ± 0.04	−52.05 ± 0.65	−47.36 ± 0.35	−0.51 ± 0.02	−4.59 ± 0.41

* The average values of all corresponding production runs are presented as mean ± standard deviation. Standard deviations were calculated from corresponding mean contributions obtained in four independent 10 ns production runs of MD simulations. ** Binding free energies were calculated with preoptimized values of α = 0.18 and β = 0.33. The experimental binding free energy of a positive control rolipram equals −9.86 kcal/mol [83].

## Data Availability

All data generated or analysed during this study are included in the published article.

## Supplementary Materials

The following are available online at https://www.mdpi.com/2218-273X/11/3/479/s1, Figure S1: The active site subpockets together with the CR3 region, metal ions, and coordinated water molecules of the PDE4D enzyme, Figure S2: Native and best-scoring redocked poses of PDE4D inhibitor 15X at the active site of PDE4D enzyme (PDB ID: 3IAD, chain A), Figure S3: Average RMSD curves of PDE4D active site atomic positions in the radius of 6 Å around the ligand throughout four 10 ns molecular dynamics simulation production runs of the four systems: curcumin in complex with PDE4D, 6-gingerol in complex with PDE4D, capsaicin in complex with PDE4D, and resveratrol in complex with PDE4D, Figure S4: Average RMSF graphs of PDE4D active site atomic positions in the radius of 6 Å around the ligand throughout four 10 ns molecular dynamics simulation production runs of the four systems: curcumin in complex with PDE4D, 6-gingerol in complex with PDE4D, capsaicin in complex with PDE4D, and resveratrol in complex with PDE4D, Figure S5: The binding modes of curcumin, 6-gingerol, capsaicin, and resveratrol in comparison with the binding modes of the native inhibitor 15X as well as the established PDE4D inhibitor rolipram.

## Abbreviations

AD	Alzheimer’s disease
Aβ	Amyloid-β peptide
Bcl-2	B-cell lymphoma 2
BDNF	Brain-derived neurotrophic factor
cAMP	Cyclic adenosine monophosphate
CNS	Central nervous system
COPD	Chronic obstructive pulmonary disease
CR3	Control region 3
CREB	cAMP response element binding protein
ele	electrostatic interactions
Epac1/2	Exchange factor directly activated by cAMP 1 and 2
ESP	Electrostatic potential
FDA	Food and Drug Administration
FEP	Free energy perturbation
GAFF	General AMBER force field
HF	Hartree-Fock
IBD	Inflammatory bowel diseases
IL-1β	Interleukin 1 beta
IL-6	Interleukin 6
LIE	Linear interaction energy
LRA	Linear response approximation
LRF	Local reaction field
MD	Molecular dynamics
PDB	Protein data bank
PDE4	Phosphodiesterase 4
PKA	Protein kinase A
RA	Rheumatic arthritis
RESP	Restricted electrostatic charge fitting procedure
PLIP	Protein ligand interaction profiler
RMR6	Radial-mean-reduced scoring function at a cutoff radius of 6 Å from each atom of the ligand
RMSD	Root mean square deviation
RMSF	Root mean square fluctuation
ROS	Reactive oxygen species
SCAAS	Surface constraint all-atom solvent
UCR1	Upstream conserved region 1
UCR2	Upstream conserved region 2
vdW	van der Waals interactions
