# Supplementary material for: Insight into Inhibitory Mechanism of PDE4D by Dietary Polyphenols Using Molecular Dynamics Simulations and Free Energy Calculations
Source: Biomolecules. 2021 Mar 23;11(3):479. doi: 10.3390/biom11030479 (PMC8004924; doi:10.3390/biom11030479)
Supplement: Supplementary file 1 [file biomolecules-11-00479-s001.pdf]

Supplementary Materials

Insight into Inhibitory Mechanism of PDE4D by Dietary Polyphenols Using Molecular Dynamics Simulations and Free Energy Calculations

Veronika Furlan[1], Urban Bren[1,2,*]

[1] Faculty of Chemistry and Chemical Engineering, University of Maribor, Smetanova 17, SI-2000 Maribor, Slovenia; veronika.furlan@um.si

[2] Faculty of Mathematics, Natural Sciences and Information Technologies, University of Primorska, Glagoljaška 8, SI-6000 Koper, Slovenia

* Correspondence: Email: urban.bren@um.si. Phone: +386-2-229 4421.

The active site subpockets together with the CR3 region, metal ions, and coordinated water molecules of the PDE4D enzyme are visualized in Figure S1.

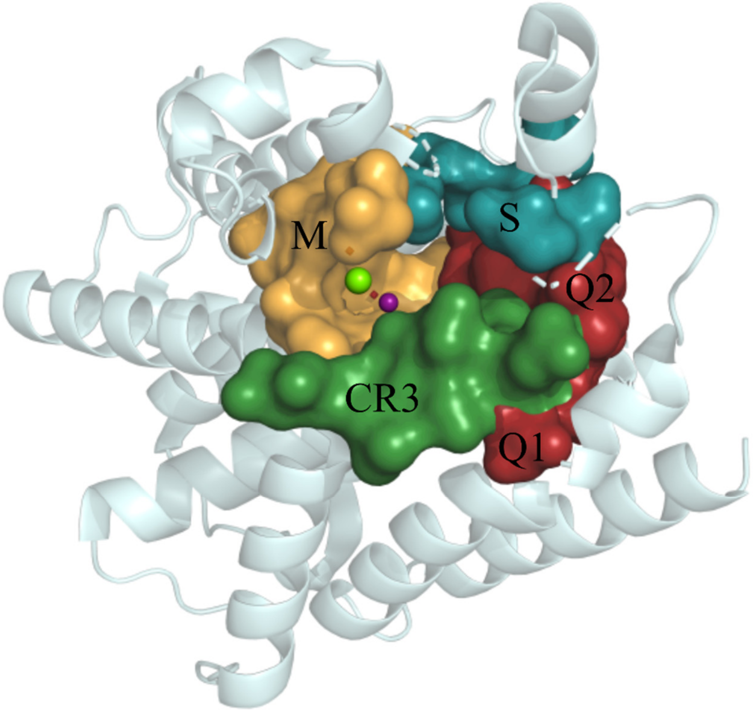

Figure S1. The active site subpockets together with the CR3 region, metal ions, and coordinated water molecules of the PDE4D enzyme. Metal pocket M is depicted in orange, solvated pocket S in blue, hydrophobic Q pocket in red, and the CR3 region in green. The magnesium ion is presented in light green and the zinc ion in violet color. The coordinated water molecules are presented as red dots.

The comparison of the native and best-scoring redocked 15X poses at the active site of the PDE4D enzyme (PDB ID: 3IAD, chain A) is presented in Figure S2.

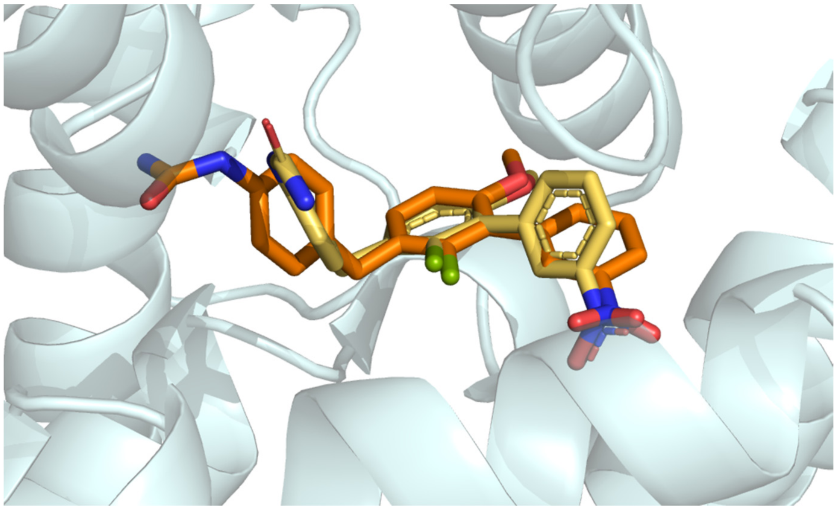

Figure S2. Native (yellow) and best-scoring redocked (orange) poses of PDE4D inhibitor 15X at the active site of PDE4D enzyme (PDB ID: 3IAD, chain A). Oxygen atoms are red, nitrogen atoms dark blue and fluorine atoms green. The calculated RMSD value between the best-scoring docked pose and the native pose of the PDE4D inhibitor 15X equals 0.94 Å and additionally confirms the validity of the applied molecular docking protocol.

Average RMSD and RMSF graphs of PDE4D active site atomic positions in the radius of 6 Å around the ligand throughout four 10 ns molecular dynamics simulation production runs of the four systems: curcumin in complex with PDE4D, 6-gingerol in complex with PDE4D, capsaicin in complex with PDE4D, and resveratrol in complex with PDE4D, are presented in Figures S3 and Figure S4, respectively.

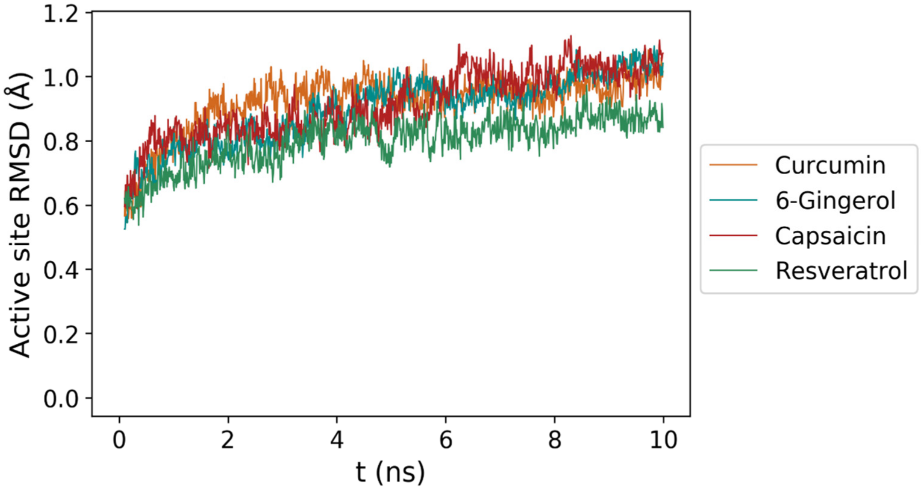

Figure S3. Average RMSD curves of PDE4D active site atomic positions in the radius of 6 Å around the ligand throughout four 10 ns molecular dynamics simulation production runs of the four systems: curcumin in complex with PDE4D (orange), 6-gingerol in complex with PDE4D (blue), capsaicin in complex with PDE4D (red), and resveratrol in complex with PDE4D (green).

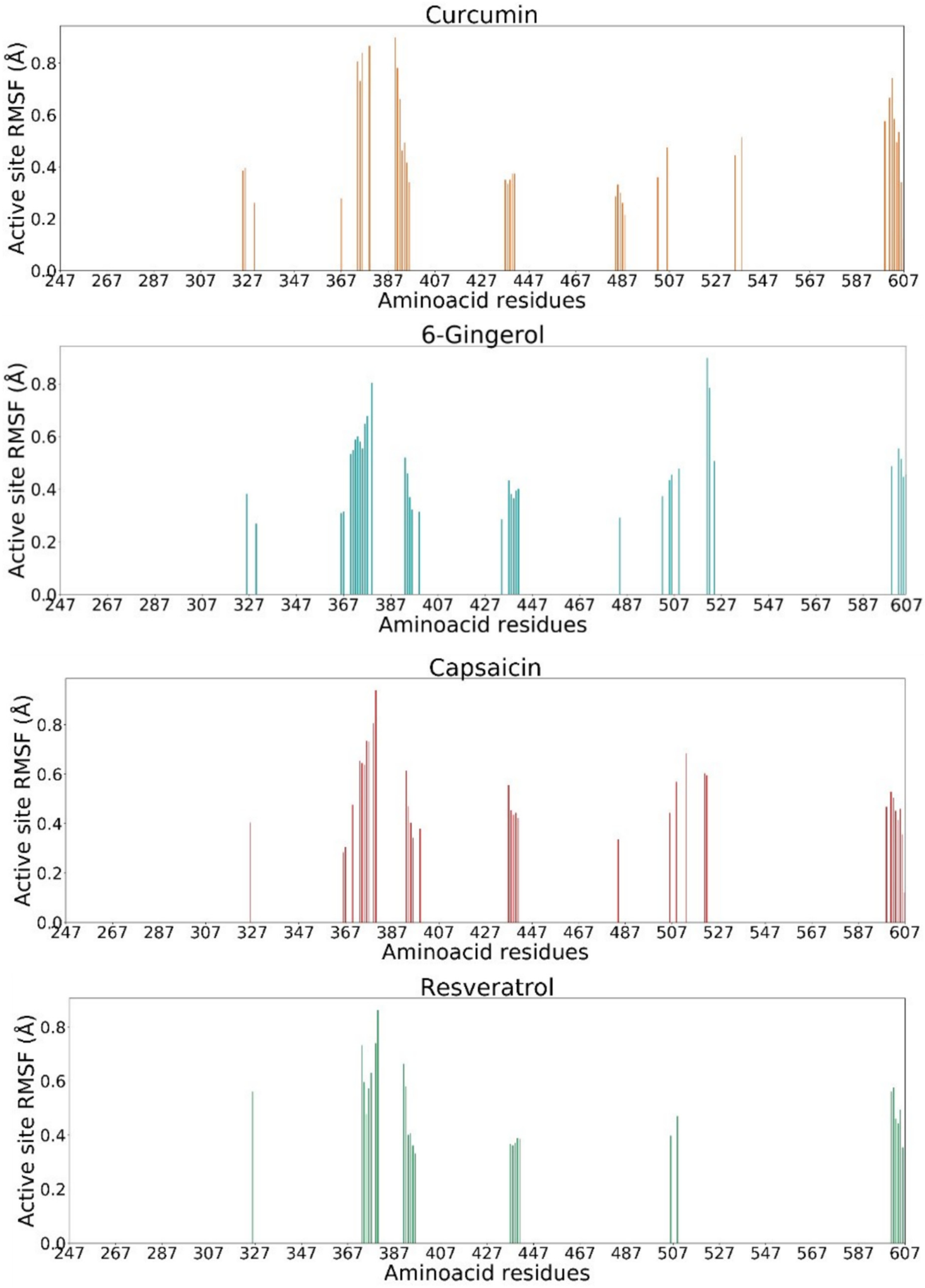

Figure S4. Average RMSF graphs of PDE4D active site atomic positions in the radius of 6 Å around the ligand throughout four 10 ns molecular dynamics simulation production runs of the four systems: curcumin in complex with PDE4D (orange), 6-gingerol in complex with PDE4D (blue), capsaicin in complex with PDE4D (red), and resveratrol in complex with PDE4D (green). It can be observed, that similar aminoacid regions play a role in the investigated natural product binding to PDE4D, which confirms that all the ligands occupy the same active site.

The graphical comparison of the binding modes of the studied polyphenols and the native inhibitor 15X as well as the established PDE4D inhibitor rolipram is presented in Figure S5.

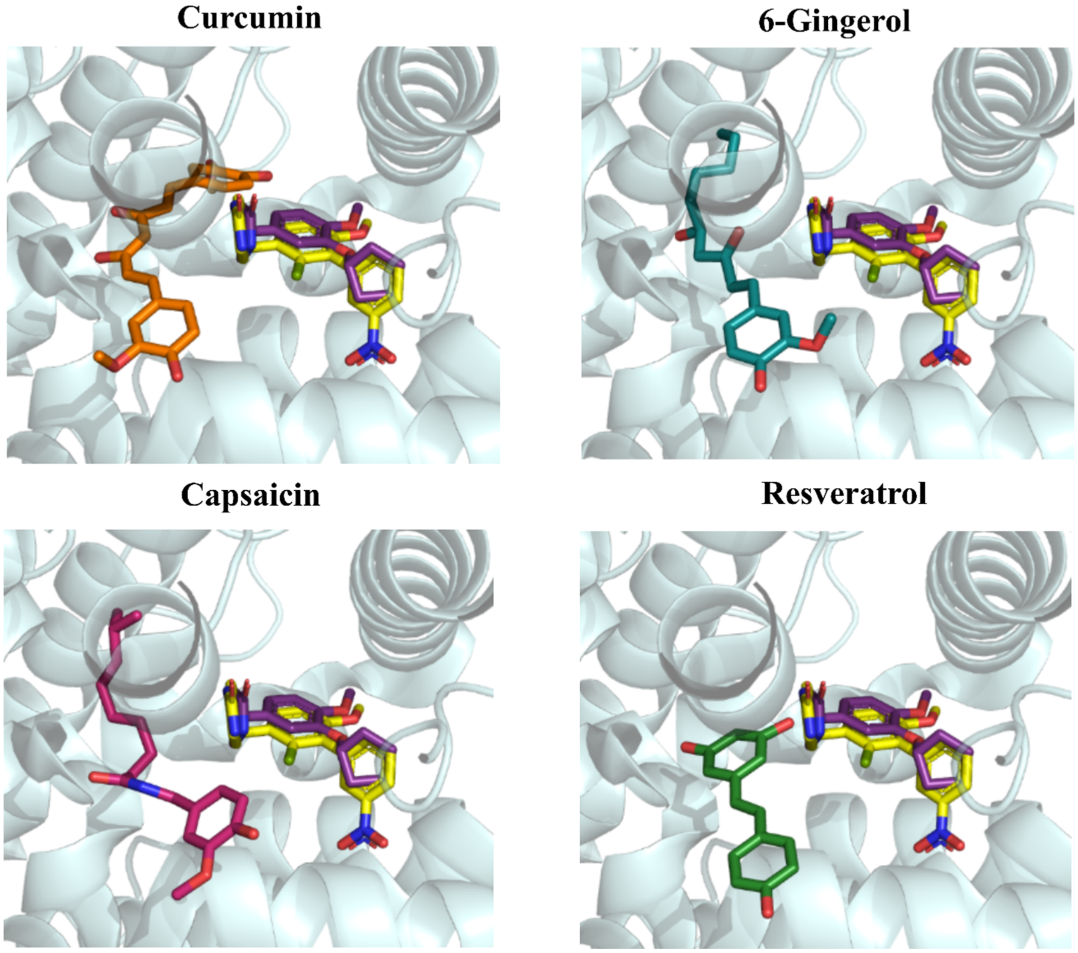

Figure S5. The binding modes of curcumin (orange), 6-gingerol (blue), capsaicin (pink), and resveratrol (green) in comparison with the binding modes of the native inhibitor 15X (yellow) as well as the established PDE4D inhibitor rolipram (violet). The rolipram structure in complex with PDE4D (PDB ID 1TBB, chain A) was after superimposition of the protein structure transferred to the studied crystal structure (PBD ID 3IAD, chain A).
